# Soil Respiration in Tibetan Alpine Grasslands: Belowground Biomass and Soil Moisture, but Not Soil Temperature, Best Explain the Large-Scale Patterns

**DOI:** 10.1371/journal.pone.0034968

**Published:** 2012-04-11

**Authors:** Yan Geng, Yonghui Wang, Kuo Yang, Shaopeng Wang, Hui Zeng, Frank Baumann, Peter Kuehn, Thomas Scholten, Jin-Sheng He

**Affiliations:** 1 Department of Ecology, College of Urban and Environmental Sciences, and Key Laboratory for Earth Surface Processes of the Ministry of Education, Peking University, Beijing, China; 2 Key Laboratory of Adaptation and Evolution of Plateau Biota, Northwest Institute of Plateau Biology, Chinese Academy of Sciences, Xining, China; 3 Shenzhen Key Laboratory of Circular Economy, Shenzhen Graduate School, Peking University, Shenzhen, China; 4 Department of Geoscience, Physical Geography and Soil Science, University of Tuebingen, Tuebingen, Germany; DOE Pacific Northwest National Laboratory, United States of America

## Abstract

The Tibetan Plateau is an essential area to study the potential feedback effects of soils to climate change due to the rapid rise in its air temperature in the past several decades and the large amounts of soil organic carbon (SOC) stocks, particularly in the permafrost. Yet it is one of the most under-investigated regions in soil respiration (Rs) studies. Here, Rs rates were measured at 42 sites in alpine grasslands (including alpine steppes and meadows) along a transect across the Tibetan Plateau during the peak growing season of 2006 and 2007 in order to test whether: (1) belowground biomass (BGB) is most closely related to spatial variation in Rs due to high root biomass density, and (2) soil temperature significantly influences spatial pattern of Rs owing to metabolic limitation from the low temperature in cold, high-altitude ecosystems. The average daily mean Rs of the alpine grasslands at peak growing season was 3.92 µmol CO_2_ m^−2^ s^−1^, ranging from 0.39 to 12.88 µmol CO_2_ m^−2^ s^−1^, with average daily mean Rs of 2.01 and 5.49 µmol CO_2_ m^−2^ s^−1^ for steppes and meadows, respectively. By regression tree analysis, BGB, aboveground biomass (AGB), SOC, soil moisture (SM), and vegetation type were selected out of 15 variables examined, as the factors influencing large-scale variation in Rs. With a structural equation modelling approach, we found only BGB and SM had direct effects on Rs, while other factors indirectly affecting Rs through BGB or SM. Most (80%) of the variation in Rs could be attributed to the difference in BGB among sites. BGB and SM together accounted for the majority (82%) of spatial patterns of Rs. Our results only support the first hypothesis, suggesting that models incorporating BGB and SM can improve Rs estimation at regional scale.

## Introduction

Soil respiration (Rs) is the major pathway for carbon (C) exiting terrestrial ecosystems and plays a central role in global carbon cycles [1]–[3]. Because soil is the largest carbon pool in terrestrial ecosystems, containing more than 1500 Pg C (1 PG = 10^15^ g) [4]–[6], small change in the rate of Rs may have a profound impact on atmospheric CO_2_ concentration, exerting positive feedbacks to global warming [2], [7]–[9]. Therefore, it is important to understand and be able to predict how Rs responds to environmental variation and climate change.

Rs has been a major research theme, particularly since the beginning of 1990s [2], [6], [10]–[16]. Many studies in a variety of ecosystems have been devoted to evaluation of various influencing factors, including microbial activity [17]–[19], C allocation [20], [21], root dynamics [22], and regulators such as temperature, soil moisture, soil texture and other climatic and soil variables [23], [24]. Nevertheless, synthetic analyses of existing data show a substantially huge heterogeneity in Rs, for which reason we require comprehensive datasets before being able to discuss the uncertainties that may arise owing to differences in intensity of sampling in different ecosystems [25].

It has been well documented that Rs varies greatly with time and space [25]. With the advanced equipment for high-frequency records of Rs, temperature, moisture and other variables (e.g. [26]), within-site temporal patterns of Rs can be relatively easily obtained. However, to address patterns of ecosystem C cycling at regional scale, to predict responses of Rs to future climate change based on mechanistic data, and to scale-up from specific sites to vegetation biomes, studies on Rs need to move beyond within-site variations in soil temperature and soil moisture and to incorporate differences among broad ecosystem types [6], [27], [28]. At regional scale, patterns of biogeochemical cycling of different ecosystem types are governed by at least five independent controls or so-called state factors, i.e. climate, parent material, topography, biota, and time [3], [29]. Hence, factors closely associated with Rs within-ecosystem and among-ecosystems are not identical. However, compared with the plenty of studies on temporal variations, relatively fewer publications have explored in-depth the regional patterns of Rs and the factors revolving around Rs process (but see [30]).

The Tibetan Plateau is one of the most under-studied regions for Rs research, despite its essential role in the global C cycles. Due to rough natural conditions, only a few studies have measured Rs. Some in alpine steppe [31], some in alpine meadow [32]–[35], and others in cropland [36]. Alpine grassland accounts for 62% of the total area of the plateau, out of which 32% is alpine steppe, and 30% alpine meadow [37]. Alpine grassland is of special interest because of the high C density [38], [39] and potential feedbacks to climate warming [40]. We previously estimated that SOC storage in the top one meter in these alpine grasslands was 7.4 Pg C, with an average density of 6.5 kg m^−2^ [39]. Moreover, the Tibetan Plateau is the largest high-altitude and low-latitude permafrost area on the earth, with over 50% of its total surface in permafrost [41], [42]. The observed rapid rises in air temperature [43], degradation of the permafrost and the associated changes in soil hydrology in the last several decades [42], [44], [45] will seriously impact the C cycles [34], [46]. The high-altitude ecosystems, low-latitude permafrost, unique vegetation composition and physiological adaptation to the extreme environments, as well as the relatively low intensity of human disturbance motivated us to focus on carbon cycle and the effects of global climate change on natural ecosystems of the Tibetan Plateau.

The primary objective of this study is to investigate large-scale spatial patterns of Rs and to examine their responses to naturally occurring environmental gradients in order to identify factors most closely associated with Rs in such extreme environments. We hypothesized that:

1. Belowground biomass is most related to large-scale variations in Rs, because alpine grasslands have a high root biomass density [47]. As a result autotrophs will contribute a large proportion of the total respiratory CO_2_ efflux.
2. Soil temperature is another important influential factor for alpine grassland Rs. This is because low growing-season temperature is a limiting factor for physiological processes in high-altitude grassland ecosystems [48], [49]. Therefore, it is predicted that Rs increases with increasing soil temperature.

These two hypothesis were tested in a transect study across alpine grasslands on the Tibetan Plateau. The measurement of Rs in this vast, remote, high-altitude area complements the existing data and help to estimate the global C flux from soils.

## Materials and Methods

### Ethics Statement

No specific permits were required for the described field studies in the Tibetan Plateau. The research sites are not privately-owned or protected in any way and field studies did not involve endangered or protected species.

### Study sites

**Figure 1:**
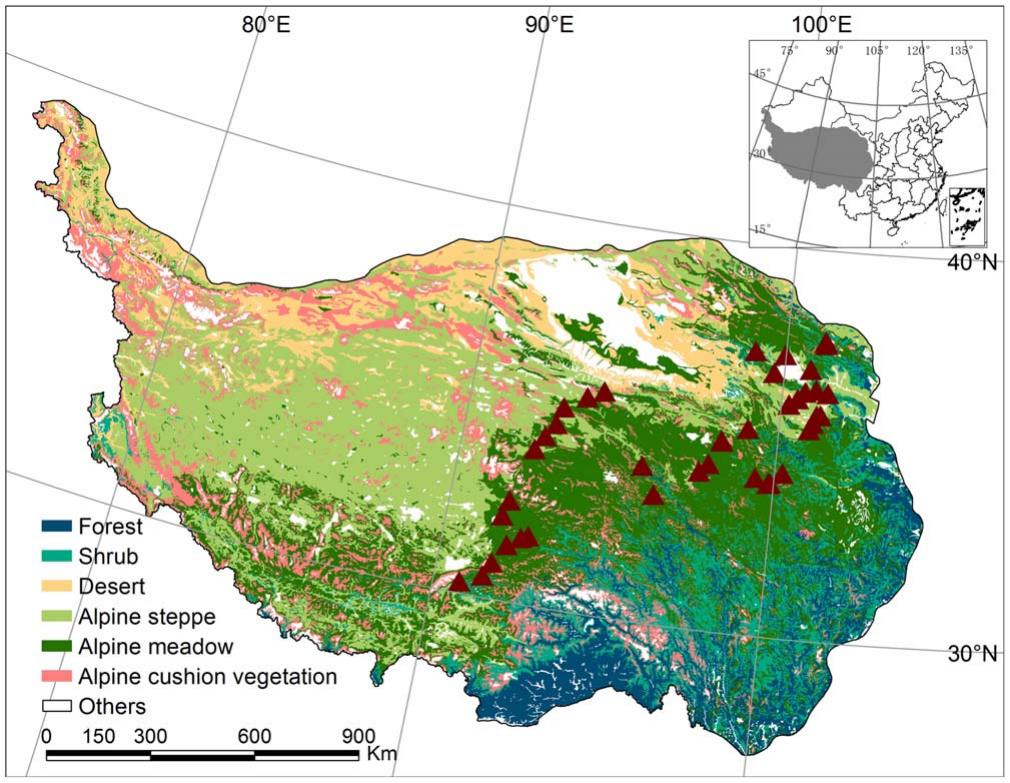
Vegetation map of the sampling sites, selected from the Vegetation Map of China [80]. Triangles represent sampling sites.

This study was conducted during two expeditions in late July and early August of 2006 and 2007, in collaboration with University of Tuebingen, Germany. Out of the 51 sites, 42 were selected for soil respiration measurements along a transect which stretches from latitudes of 30.31 to 37.69°N and longitudes of 90.80 to 101.48°E, and elevations from 2925 to 5105 m a.s.l. (Table 1, Fig. 1). Mean annual air temperature (MAT) and mean annual precipitation (MAP) range from −5.75 to 2.57°C and 218 to 604 mm yr^−1^, respectively. The vegetation represents alpine grassland, including the two main ecosystem types, alpine meadow and alpine steppe [49], [50]. Out of the 42 sites, 23 were alpine meadows and 19 alpine steppes. Alpine meadows are dominated by perennial tussock grasses such as *Kobresia pygmaea* and *K. tibetica*, while alpine steppes are dominated by short and dense tussock grasses such as *Stipa purpurea*; both ecosystem types have extensive distributions. The sites were selected by visual inspection of the vegetation, aiming to sample sites subject to minimal grazing and other anthropogenic disturbances.

**Table 1:** Description of 42 sites where soil respiration measurements were taken.

Site	Latitude	Longitude	Altitude (m)	MAT (°C)	GST (°C)	MAP (mm yr^−1^)	GSP (mm yr^−1^)	Rs (µmol m^−2^ s^−1^)	Vegetation
QZ01	36.37	101.48	3454	−1.83	7.33	466	326	4.35	Meadow
QZ02	35.80	101.30	3302	0.03	8.98	475	328	5.27	Meadow
QZ03	35.78	101.17	3263	0.39	9.37	466	322	3.26	Steppe
QZ04	35.58	101.08	3416	−0.37	8.50	488	336	2.87	Steppe
QZ06	35.41	100.97	3517	−0.79	7.99	501	346	4.07	Meadow
QZ07	34.24	100.25	4282	−4.23	3.96	604	414	5.09	Meadow
QZ08	33.96	99.88	4053	−2.11	6.06	580	395	4.08	Meadow
QZ11	33.94	99.83	4156	−2.77	5.38	589	402	5.15	Meadow
QZ13	34.06	99.40	4231	−3.22	5.05	568	389	12.9	Meadow
QZ14	34.92	98.21	4267	−3.96	4.96	464	326	5.06	Meadow
QZ15	34.89	98.23	4224	−3.63	5.27	462	325	2.14	Steppe
QZ17	34.28	97.88	4667	−5.74	2.84	522	364	9.32	Meadow
QZ18	33.32	96.28	4506	−2.89	5.48	482	333	6.00	Meadow
QZ19	34.01	95.80	4201	−1.60	7.15	390	274	3.58	Steppe
QZ22	34.06	97.60	4700	−5.55	2.97	523	365	2.46	Meadow
QZ23	35.29	99.01	4217	−4.48	4.47	478	336	1.19	Steppe
QZ24	36.01	100.25	3109	1.63	10.92	393	274	1.59	Steppe
QZ25	36.17	100.51	2925	2.57	11.93	380	264	0.89	Steppe
QZ26	36.36	100.74	3233	0.08	9.43	409	287	2.19	Steppe
QZ27	36.44	101.09	3486	−1.94	7.32	446	314	5.36	Meadow
QZ28	36.95	100.86	3130	−0.01	9.62	372	265	2.52	Steppe
QZ29	37.26	99.98	3215	−0.55	9.39	319	233	1.78	Steppe
QZ30	37.28	98.99	3437	−1.61	8.48	290	216	4.04	Steppe
QZ31	35.74	94.25	4222	−3.14	6.70	218	170	2.17	Steppe
QZ32	35.52	93.74	4564	−5.01	4.80	238	185	0.39	Steppe
QZ33	35.17	93.04	4682	−5.41	4.31	234	182	0.75	Steppe
QZ34	34.72	92.89	4801	−5.75	3.76	348	249	4.23	Meadow
QZ35	33.99	92.35	4654	−4.22	4.94	336	248	1.24	Steppe
QZ36	32.18	91.72	4903	−4.18	4.12	473	327	2.79	Meadow
QZ38	31.45	92.02	4494	−0.25	7.94	480	341	1.12	Meadow
QZ40	31.77	92.62	4605	−2.05	5.89	523	361	3.03	Meadow
QZ41	31.69	92.41	4596	−1.92	6.00	511	355	3.99	Meadow
QZ42	30.94	91.66	4756	−2.76	5.45	539	371	1.90	Steppe
QZ43	30.56	91.45	4506	−0.53	7.32	507	359	5.94	Meadow
QZ44	30.31	90.80	4324	1.23	8.81	442	326	1.99	Steppe
QZ45	32.58	91.86	5105	−5.75	2.77	488	331	4.59	Meadow
QZ46	34.37	92.61	4656	−4.56	4.78	327	241	1.78	Steppe
QZ47	36.78	99.67	3391	−1.00	8.72	348	251	8.25	Meadow
QZ48	37.61	101.31	3196	−1.53	7.74	363	309	7.26	Meadow
QZ49	37.61	101.31	3196	−2.12	7.19	364	311	10.6	Meadow
QZ50	37.69	101.28	3268	−1.89	7.67	313	270	5.32	Meadow
QZ51	37.28	98.99	3437	−1.61	8.48	290	216	1.99	Steppe

MAT, Mean annual temperature; GST, growing season temperature; MAP, mean annual precipitation; GSP, growing season precipitation; Rs, daily mean soil respiration rate.

### Field measurements

At each site, we conducted (1) measurement of plant biomass after surveying the entire plant community, (2) collections of soil samples at three depths (0–5, 5–10, and 10–20 cm) using soil corer, followed by volumetric samples at equal depths for bulk density and gravimetric water content determinations, (3) on-site extraction of soil mineralized N (N_min_) consisting of nitrate (NO_3_-N) and ammonium (NH_4_-N), and (4) measurement of soil respiration rates.

#### Plant biomass measurement

We harvested aboveground biomass (AGB) in three plots (1×1 m^2^) and belowground biomass (BGB) in three soil pits (0.5×0.5 m^2^) described in Yang et al. [47]. Biomass samples were dried using a custom-built portable oven in the field, and oven-dried at 60°C to a constant weight, and weighed to the nearest 0.01 g upon returning to the laboratory.

#### Soil property measurement

Soil sampling procedures, soil bulk density (SBD), soil total N (STN) and SOC measurements have been detailed elsewhere [39]. On-site extraction of N_min_ was carried out using a custom-designed equipment which could perform on-site extraction without any disturbances. In brief, 10 g of homogenized soil was extracted with 50 ml 1 mol KCl for 60 minutes immediately after sampling, filtered through Whatman No. 42 cellulose filter paper into 100 ml PE-vials, and conserved by acidification with 3 ml hydrochloric acid (HCl, 30%) [38].

#### Soil respiration measurement

**Figure 2:**
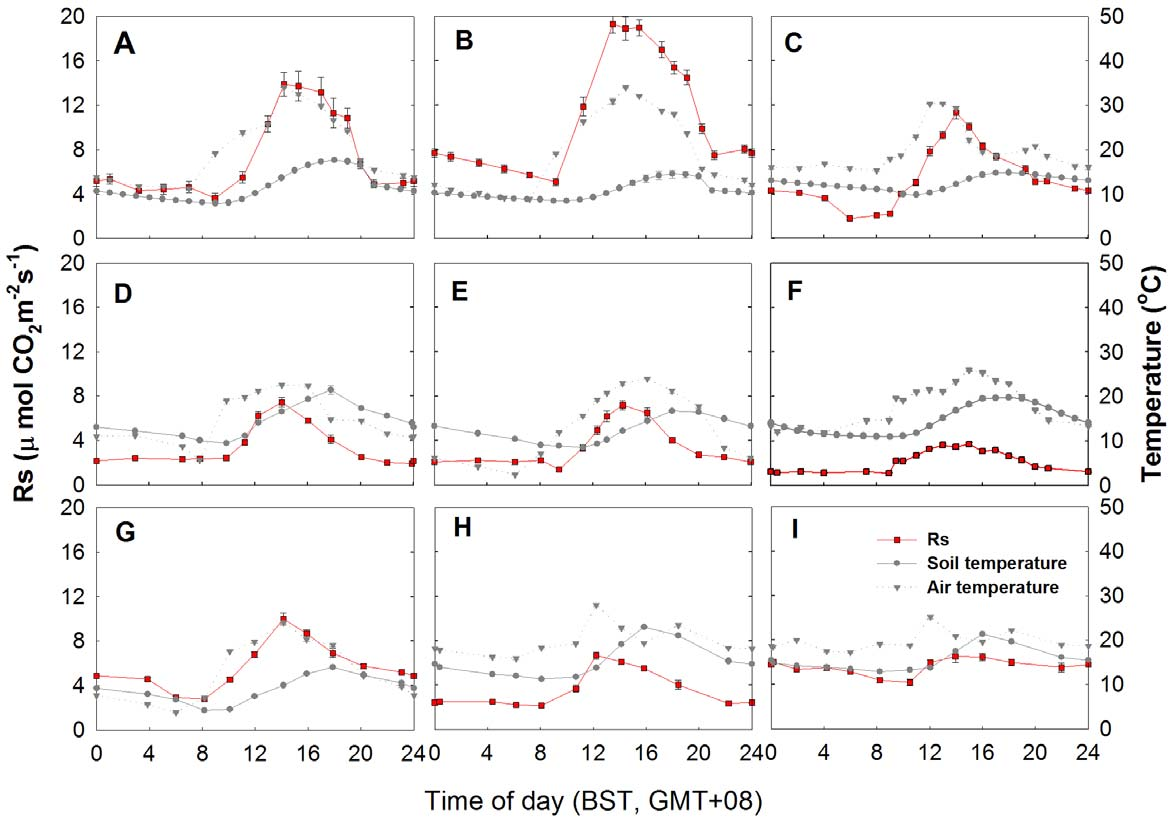
Diurnal changes of soil respiration rate, soil temperature and air temperature. Complete diurnal courses of soil respiration were measured for seven alpine meadows and two alpine steppes on the Tibetan Plateau. Vertical bars indicate the standard error of the measurement mean (n = 5–7) for each time. (A), Haibei, *Kobresia* and *Festuca* mixed meadow; (B), Haibei, *Kobresia tibetica* meadow; (C), Haibei, *Kobresia pygmaea* meadow; (D) Naqu, *Kobresia pygmaea* meadow; (E) Naqu, *Kobresia tibetica* meadow; (F) Tianjun, *Stipa purpurea* steppe; (G) Fenghuoshan, *Kobresia pygmaea* meadow; (H) Qumalai, *Kobresia pygmaea* meadow; (I) Qumalai, *Festuca* steppe.

At each site, seven PVC soil collars (10 cm inside diameter and 5 cm in height) were installed 2–3 cm into the soil along a straight line at one-meter intervals. Rs (CO_2_ efflux) was measured with a Li-6400 infrared gas analyzer equipped with the 6400-09 soil flux chamber (Li-Cor Inc, Lincoln, NE, USA). The protocol recommended by LiCor (LI-6400-09 manual) was changed to five observations of 10 µmol mol^−1^ (for steppes) and 30 µmol mol^−1^ (for meadows) per measurement. Typically, soil respiration rates were measured 3–4 times during 4–5 hours from 10:00 to 16:00 (Beijing Standard Time) when soil respiration peaked. To obtain the diurnal pattern, we also measured the complete diurnal variation of soil respiration at nine sites (Fig. 2). We then calculated the ratios of instant Rs from 10:00 to 16:00 to the daily mean Rs for the nine sites. Using these ratios, we calculated daily mean Rs of non-diurnal sites according to similarity in community composition and closeness in distance. On average, diurnal courses of soil respiration were measured every four to five sites. Soil temperature at 10 cm was monitored simultaneously with soil respiration measurement using the attached soil temperature probe. Air temperature was measured with the temperature probe of Li-6400 infrared gas analyzer.

### Laboratory analysis

Dried soil samples were grounded using a ball mill (NM200, Retsch, Germany). Total C and N concentrations were determined on 5–6 mg aliquot of the homogenously grounded material for each sample using an elemental analyzer (2400 II CHNS/O Elemental Analyzer, Perkin-Elmer, Boston, MA, USA) with a combustion temperature of 950°C and a reduction temperature of 640°C. Soil inorganic carbon (SIC) was measured volumetrically using an inorganic carbon analyzer (Calcimeter 08.53, Eijkelkamp, Netherland). Thus SOC was calculated as the difference between STC and SIC. Soil pH was determined in both 0.01 M CaCl_2_ and bi-distilled H_2_O potentiometrically, but only those of water solution were used in the current study. The KCl-extractions for N_min_-analysis were measured photometrically using a Continuous Flow Analyzer (SAN Plus, Skalar, Netherlands). Soil moisture (SM) was determined gravimetrically by taking the skeleton content into account.

### Climate data and statistical analysis

At each site, we installed temperature data loggers (Hobo U12, Onset Computer Corporation, Pocasset, MA) in July 2006 to measure soil temperature (−10 cm) at 1 h interval. We revisited these sites in July or August in 2007, 2008 and 2009 to download the recorded temperature data. Based on those measurements mean annual soil temperature (MAST) of each site was calculated. The climate data used in this study were calculated based on linear models using latitude, longitude, and altitude as variables from 55-year averaged temperature and precipitation records (1951–2005) at 680 well-distributed climate stations across China [48], [51], [52]

The variables to explain the spatial variation of soil respiration consist of (1) soil properties, measured by SOC, SM, MAST, soil C/N ratio, SBD, soil acidity (pH), soil texture (sand content, clay content), and N_min_, (2) average climate, encompassing growing season temperature (GST), growing season precipitation (GSP), and (3) plant community characteristics, including vegetation type (VT, meadow or steppe), AGB and BGB (Table 2). We used regression tree analysis [53], as implemented in the SAS statistical software package version 8.01 [54], to screen important variables influencing soil respiration, as tree-based modeling is an exploratory data analytic technique for summarizing multivariable and uncovering its structure in large datasets [55]. We selected *F* test's *p*-value as splitting criterion, and set observations required for a split search at 5. Our sample size (42 sites) doesn't allow us to do cross validation, but when we set the *F* test significant level at 0.20, the tree developed was adequate in complexity (depth) and explanation (*R* ^2^). From the relative importance in the regression tree which was calculated as the cumulative variance reduction at each split for a particular independent variable, five variables with the importance values greater than 0.4 were screened out, i.e. AGB, BGB, VT, SOC, and SWC (Table 2).

**Table 2:** Variables included in the regression tree analysis and their importance value.

Variable	n	Mean	SD	Range	Importance in regression tree
Soil organic carbon (SOC, %)	42	5.25	4.79	0.339–19.4	1.0000
Aboveground biomass (AGB, g m^−2^)	42	119	100	29.9–530	0.8997
Belowground biomass (BGB, g m^−2^)	42	1816	1957	202–9393	0.8889
Vegetation type (VT)	42	-	-	-	0.4577
Soil moisture (SM, v/v, %)	42	38.3	50.2	0.44–220	0.4383
Growing season temperature (GST, °C)	42	6.67	2.25	2.77–11.93	0.1719
Mean annual soil temperature (MAST, °C)	42	17.0	5.53	−1.12–8.14	0.1621
Growing season precipitation (GSP, mm yr^−1^)	42	306	61.5	170–414	0.0000
Soil temperature (ST, °C)	42	17.0	5.53	6.30–31.55	0.0000
Soil C/N ratio (C/N, g g^−1^)	39	12.1	2.85	7.97–20.1	0.0000
Soil bulk density (SBD, g cm^−3^)	38	0.94	0.32	0.31–1.65	0.0000
pH	38	7.3	0.52	6.0–8.1	0.0000
Sand content (%)	37	42.3	18.4	20.0–80.0	0.0000
Clay content (%)	37	7.60	6.59	3.0–24	0.0000
Available nitrogen (mmol l^−1^)	37	0.080	0.046	0.026–0.218	0.0000

To address how these variables affect soil respiration both directly and indirectly is challenging because variables measured in field are cross-correlated [11], [14], [28]. Structural equation modelling (SEM) [56]–[58] has been used in recent studies to explicitly evaluate the causal relationships among multiple interacting variables (e.g. [59]–[61]). SEM aims to account for the roles of multiple variables in a single analysis, providing mechanisms behind the overall patterns by partitioning direct from indirect effects that act through other components of the system.We used SEM here to partition the total effect of variables on soil respiration into direct effects and indirect effects. A path model was developed to relate soil respiration to AGB, BGB, VT, SOC, and SWC, based on theoretical knowledge of the major factors associated with soil respiration at ecosystem level [3]. The model was fitted using EQS 6.1 for Windows [62].

As the results of SEM are dependent on correctly specifying theoretical causal relationships between variables prior to analysis [56], [58], the initial theoretical model was modified to improve the fit between model and data. The final model was strong: Bentler's comparative fit index (CFI) = 0.96, Bentler-Bonett normed fit index (NFI) = 0.95. Furthermore, R-squares for Rs, AGB, BGB are very high in the path model.

## Results

### Overall soil respiration

Across 42 sites, the daily mean Rs of alpine grassland at peak growing season was 3.92 µmol CO_2_ m^−2^ s^−1^, and ranged from 0.39 to 12.88 µmol CO_2_ m^−2^ s^−1^ (Table 1), with a coefficient of variation (CV) of 69.1%. The daily mean Rs of steppes was 2.01 µmol CO_2_ m^−2^ s^−1^ (ranged from 0.39 to 4.04), while Rs of meadows, 5.49 µmol CO_2_ m^−2^ s^−1^ (ranged from 1.12 to 12.88), was approximately two and half times that of the steppes. Although the meadows had a significantly higher Rs than steppes, their CV were similar, being 48.9 and 47.1% for meadow and steppe, respectively.

Large diurnal variations in Rs were observed, although the diurnal patterns were generally similar for meadow and steppe (Fig. 2), both exhibiting the highest Rs during the time from 12:00 to 14:00 BST. Rs and their climatic, community and soil properties for the important ecosystem types, such as *Kobresia pygmaea* meadow, *K. tibetica* meadow, species-rich meadow (mixed-species meadow), and *Stipa* spp. steppe are lised in Table 3. *K. tibetica* meadow had the highest Rs, while *Stipa* steppe had the lowest Rs.

**Table 3:** Soil respiration, community biomass, soil properties, and climatic variables in different ecosystem types.

Variable	*n*	Mean	SD	*n*	Mean	SD	*n*	Mean	SD	*n*	Mean	SD	*n*	Mean	SD
	*Kobresia pygmaea* meadow			*Kobresia tibetica* meadow			*Stipa* spp. steppe			Mixed-specie meadow			Others		
Rs (µmol m^−2^ s^−1^)	11	4.36	1.52	4	9.34	3.49	12	2.18	0.95	3	6.93	1.52	12	2.68	1.49
SOC (%)	11	6.23	3.61	4	11.51	2.54	12	1.96	1.07	3	8.82	6.50	12	4.66	5.68
AGB (g m^−2^)	11	107	65	4	285	188	12	78	40	3	253	114	12	84	41
BGB (g m^−2^)	11	2390	1261	4	5852	2682	12	528	272	3	3299	2051	12	862	619
SM (g water g^−1^ dry soil)	11	32.2	20.4	4	125.1	26.3	12	7.0	4.2	3	45.4	37.7	12	44.6	68.3
GST (°C)	11	6.28	1.46	4	4.46	2.10	12	7.61	2.20	3	8.48	0.66	12	6.38	2.64
Soil MAT ((°C))	11	3.37	1.61	4	0.91	1.88	12	3.80	2.36	3	3.66	0.31	12	2.72	3.09
GSP (mm yr^−1^)	11	351	42	4	349	35	12	257	55	3	296	40	12	301	59
C/N (g g^−1^)	10	13.75	3.20	3	15.19	0.77	12	10.36	1.16	2	11.79	1.56	12	11.81	3.10
SBD (g cm^−3^)	10	0.79	0.20	3	0.44	0.01	12	1.14	0.24	2	0.74	0.20	12	1.00	0.35
pH	8	6.9	0.59	3	6.8	0.32	12	7. 7	0.26	3	7.4	0.09	12	7.3	0.53
Sand content (%)	10	34.6	6.0	3	31.0	4.5	12	55.7	23.7	2	37.5	9.2	10	36.1	13.5
Clay content (%)	10	8.2	6.0	3	4.0	3.8	12	5.1	3.7	2	12.5	12.0	10	9.4	8.7
Available N (mmol l^−1^)	10	0.11	0.05	3	0.12	0.03	11	0.05	0.02	2	0.15	0.02	11	0.06	0.04

Rs, daily mean soil respiration rate; SOC, soil organic carbon content; AGB, above-ground biomass; BGB, below-ground biomass; SM, soil moisture; GST, growing season temperature; MAT, Mean annual temperature; GSP, growing season precipitation; SBD, soil bulk density; n, number of sampling sites; SD, standard deviation.

### Factors associated with spatial variations in soil respiration

Based on regression analysis, five variables with an importance value greater than 0.4383 were selected (Table 2), and thus were included in the development of the structural equation models. Other variables had negligible or no impact on soil respiration.

**Figure 3:**
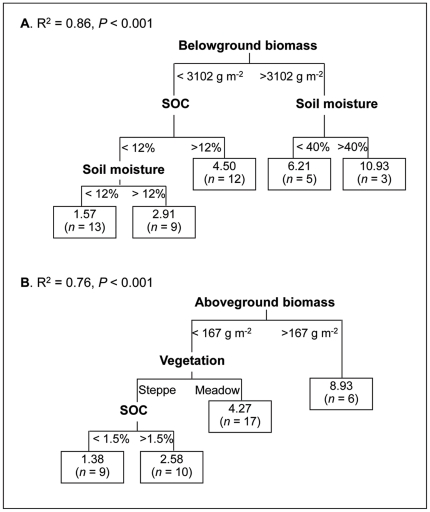
Regression tree showing generalized relationships between daily mean soil respiration rate and environmental variables. Relationships between soil respiration rate and belowground biomass, soil organic carbon content (SOC) and soil moisture (A), aboveground biomass, vegetation type, and SOC (B). Branches are labelled with criteria used to segregate data. Values in terminal nodes represent mean soil respiration rate of sites grouped within the cluster. The tree explained 86% (A) and 76% (B) of the variance in soil respiration rate, which is significantly more than a random tree (*P*<0.001). *n* = number of plots in the category.

When all five variables were entered into the model, a tree with AGB, vegetation type, and SOC as explanatory variables was developed (Fig. 3B), while BGB and SM were excluded from the model because of the close correlations between BGB and AGB, and SM and vegetation type. When BGB and SM were entered into the model, another tree was developed (Fig. 3A). Both trees are significantly more than a random tree (*P*<0.001), explaining 86% (Fig. 3A) or 76% (Fig. 3B) of the variance in Rs rate.

These analyses indicated that BGB, SOC, SM, AGB, and vegetation types are biotic and abiotic factors that are most closely associated with large-scale variations in soil respiration. For the first tree (Fig. 3A), in the areas with BGB>3102 g m^−2^, only SM had a statistically significant influence on soil respiration rate; while in the areas with BGB<3102 g m^−2^, both SOC and SM had a detectable effect. For the second tree (Fig. 3B), when AGB>167 g m^−2^, soil respiration rate was not significantly affected by vegetation type or SOC; by contrast, when AGB<167 g m^−2^, soil respiration rate was influenced by both vegetation type and SOC.

### Structural equation modelling to explain variations in soil respiration

**Figure 4:**
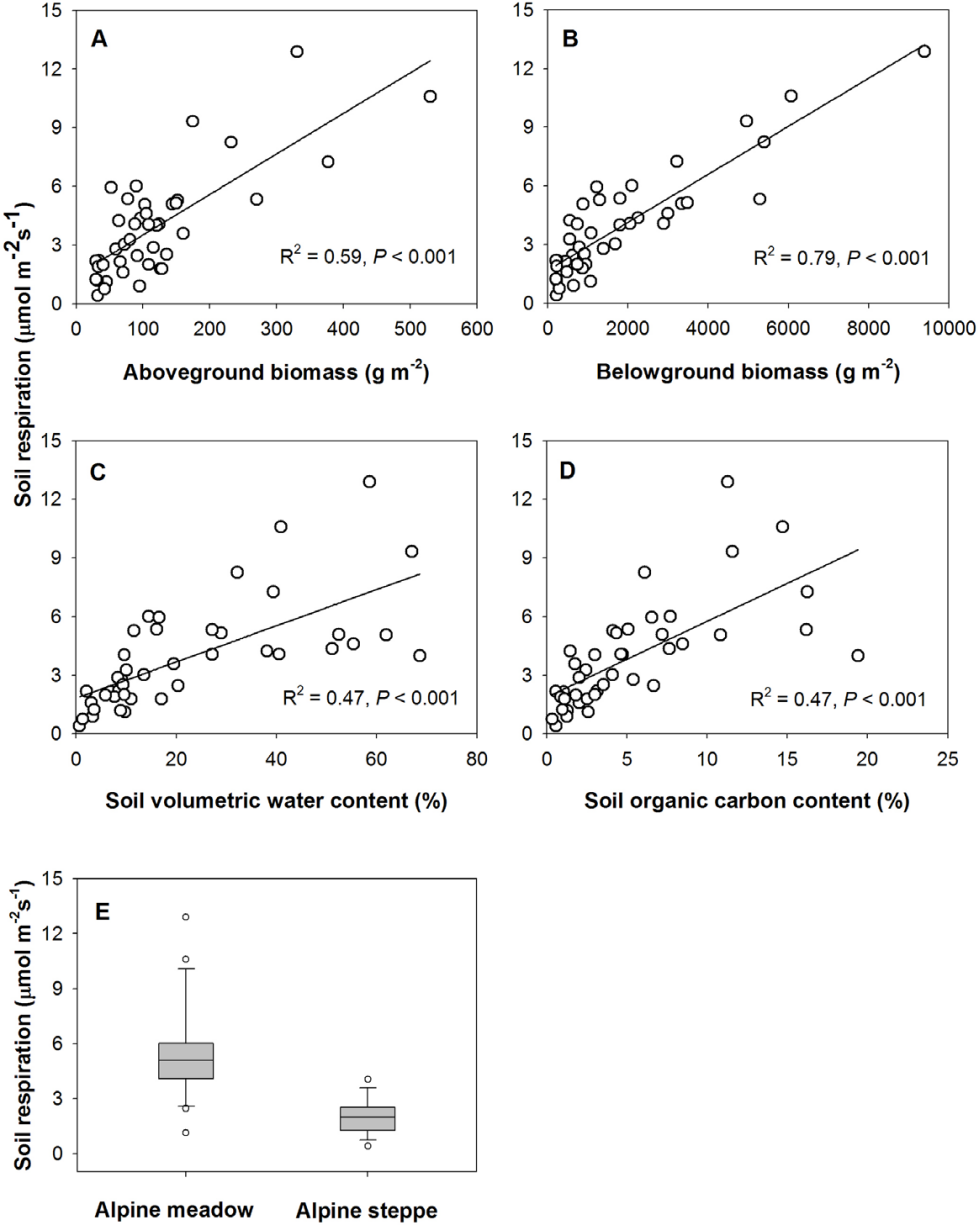
Scatterplots and box plot for daily mean soil respiration rate versus biotic and abiotic factors. Relationships between soil respiration rate and aboveground biomass (A), belowground biomass (B), soil moisture (C), soil organic carbon content (D), and vegetation type (E).

From the scatter plots and the box plot (Fig. 4), each of the selected variables such as AGB, BGB, SM, SOC and vegetation type was closely related to Rs. However, because these five variables were intercorrelated, these apparent relationships combined both direct and indirect correlations. Thus, we further used SEM to explicitly evaluate the causal relationships among these interacting variables.

**Figure 5:**
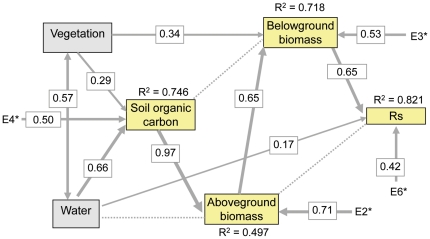
Final structural equation model for soil respiration. Non-significant paths are showed in dashed lines. The thickness of the solid arrows reflects the magnitude of the standardized SEM coefficients. Standardized coefficients are listed on each significant path.

The final SEM explained 82.1% of the variation in Rs (Fig. 5). Direct, indirect and total effects of the variables are summarized in Table 4. Increasing BGB and SM were strongly associated with increases in Rs, indicating that Rs could be well-predicted from these two variables (*R* ^2^ = 0.82). Even though there were significant bivariate relationships between AGB, SOC and Rs, they only had strong indirect positive effects on RS. Vegetation type had only an indirect effect on Rs (0.379) through its direct effect on BGB and its indirect effect on SOC and AGB. The rank of total effects, in decreasing order, was: BGB, AGB, SOC, vegetation type, and SM (Table 4).

**Table 4:** Total direct and indirect effects in the structural model. Effects were calculated using standardized path coefficients.

Variable	Direct effect	Indirect effect	Total
**Rs**			
Belowground biomass	0.654	-	0.654
Aboveground biomass	0.191 ns	0.427	0.618
Soil organic carbon	-	0.586	0.586
Vegetation type	-	0.397	0.397
Soil moisture	0.165	0.175 ns	0.335
**Belowground biomass**			
Aboveground biomass	0.652	-	0.652
Soil organic carbon	−0.021 ns	0.634	0.613
Vegetation type	0.345	0.179	0.524
Soil moisture	-	0.175 ns	0.175 ns
**Aboveground biomass**			
Soil organic carbon	0.971	-	0.971
Vegetation type	-	0.283	0.283
Soil moisture	−0.355 ns	0.644	0.29
**Soil organic carbon**			
Vegetation type	0.292	-	0.292
Soil moisture	0.663	-	0.663

Nonsignificant effects are indicated by “ns”.

It is also evident that, from the SEM (Fig. 5), BGB can well be predicted from vegetation type and AGB, explaining 71.8% of the variation. Moreover, SOC explained about 50% of the variations in AGB.

## Discussion

One common feature of natural grasslands is the climate, usually characterized by periodic droughts [63]. For a specific region, it may also be associated with basic parameters such as soil characteristics, frequent fires, grazing pressure and human activities. Chinese grasslands are generally distributed in three different regions: temperate grassland on the Inner Mongolian Plateau, alpine grassland on the Tibetan Plateau, and mountain grassland in the Xinjiang mountain areas [64]. Tibetan alpine grasslands, which are associated with cold climate of the high altitudes [49], differ from tropical and temperate grasslands. Yet, they are poorly documented in C cycles. Our survey on the large-scale patterns of Rs was preliminary, but the trend and relationships were clear.

### Magnitude of soil respiration of alpine grasslands

Large differences were observed between Rs from two vegetation types, alpine meadow and alpine steppe, being about two and half times greater in the alpine meadows. The daily mean Rs rates measured in alpine meadows (5.49 µmol CO_2_ m^−2^ s^−1^ by daily average) are similar to previously reported results. For example, Cao et al. [32]reported that during peak growing season (Mid-July or August), daily Rs was 4.4 and 3.2 µmol CO_2_ m^−2^ s^−1^ for light and heavy grazed meadows on the north-eastern edge of the Plateau. Li and Sun [33] reported a range of Rs from 0.93 to 8.02 µmol CO_2_ m^−2^ s^−1^ during growing season in their recently published results. However, the only study from the alpine steppe by Zhang et al. [31], with a daily mean Rs rate of 0.38 µmol CO_2_ m^−2^ s^−1^ at peak growing season, and an annual mean soil respiration rate of 0.248 µmol CO_2_ m^−2^ s^−1^ using a closed static chamber-gas chromatograph method in a *Stipa purpurea* and *Carex moocroftii* community, was at the lower end of our measurement. The Rs rates of alpine steppe from this study (2.01 µmol CO_2_ m^−2^ s^−1^ by daily average) are similar to the temperate steppe on the Inner Mongolia Plateau [23], [65]–[69].

Consequently, the question arises: why is there such a difference in Rs rates between the two main grassland types, alpine meadow and alpine steppe? We suggest biological differences in standing biomass and productivity as well as physical differences in soil water availability were the major factors affecting Rs. On average, AGB (proxy of aboveground productivity) and BGB of the typical *Kobresia* meadows were much greater than the typical *Stipa* steppe (Table 3). Furthermore, SM of alpine meadow was also much higher than *Stipa* steppe. These high BGB and SM in alpine meadows significantly increased Rs rate.

The alpine grasslands of the Tibetan Plateau are sometimes called alpine tundra, despite their different species composition and environmental conditions compared to arctic tundra. Nevertheless, Tibetan alpine grassland and arctic tundra share some common features, such as large below ground standing biomass (averaging 1658 g m^−2^ for arctic tundra in Alaska [70], and 1816 g m^−2^ on the Tibetan Plateau in current study), relatively large soil C density [39], [40], relatively high soil moisture (particularly in alpine meadow), and influences of permafrost. These characteristics mean that they are more responsive to global warming than other ecosystems, because their soils have the potential to release significant amounts of carbon-based greenhouse gases [46], [71], [72].

### Factors associated with the large-scale patterns of soil respiration

Our analysis showed that among biotic and abiotic factors, BGB and SM together well explained the spatial patterns of peak growing-season Rs, accounting for 82% of the variation among 42 sampling sites. The important role of SM for Rs is in good accordance with results from other studies on soil nitrogen and carbon contents across the Tibetan Plateau [39]. Most of the variation in Rs could be attributed to the difference in BGB among sites (80%), with a small proportion further explained by SM (2%, SM entered after BGB in general linear models, because BGB and SM covaried). Thus, the results support our first hypothesis that BGB is most closely associated with the large-scale variations in Rs. This finding implies that autotrophic Rs (including plant roots and closely associated organisms) contributes a large proportion to total Rs, or/and autotrophic Rs is strongly related to heterotrophic Rs in these alpine grassland ecosystems.

A few studies with data compilation have addressed the general patterns of Rs across biomes. For example, on a global scale, Raich & Schlesinger [6] found Rs is positively correlated with MAT and MAP, as well as a close correlation between mean annual net primary productivity (NPP) of different vegetation biomes and their mean annual Rs. Bond-Lamberty & Thomson [25] built a global database of Rs from 3379 records spanning publication years 1963–2008, and found MAT, MAP and leaf area index together explained approximately 41% of the observed variability in annual Rs. Across the northern hemisphere, Hibbard et al. [28] found Rs and soil temperature are closely correlated for the deciduous and mixed forests, but not for non-forest biomes. These across-biome patterns of Rs are generally controlled by climate and NPP. Furthermore, Mahecha et al. [73] approximated the sensitivity of terrestrial ecosystem respiration to MAT across 60 sites worldwide, and offers substantial evidence for a general temperature sensitivities of soil respiration. Within the grassland biome, aboveground net primary productivity (ANPP, approximation to AGB of peak growing season as in this study) was shown to be positively correlated with Rs rate [12]. Craine et al. [27] also reported in Minnesota grasslands that both AGB and BGB are positively correlated with Rs. These previous studies in grasslands are consistent with the current results, since we observed a positive correlation between AGB, BGB and Rs as well. The novel part of our study is that we found only BGB and SM had direct effects on Rs at regional scale, with other factors indirectly affecting Rs through BGB or SM. It is also evident that factors most closely associated with Rs within-biome and across biomes are different.

In contrast, intra-annual variation in Rs at individual sites are mainly explained by soil temperature and soil moisture, but not by ANPP or AGB [74]. Temporal variations of Rs have been well simulated by using the continuous records of temperature and moisture [75]. Our measurements, across altitudes from 2925 to 5105 m and mean soil temperature (−10 cm) of midday (10:00 to 16:00 BST) from 6.3 to 31.6°C (the highest soil temperature of 31.6°C was recorded in an alpine steppe at 2925 m) during the field measurement exhibited that soil temperature did not have a strong effect on Rs across study sites. For example, *Kobresia tibetica* meadow on permafrost with a soil temperature of 6.3°C still had a daily mean Rs rate as high as 5.1 µmol CO_2_ m^−2^ s^−1^. Our results from the Tibetan grassland do not support the second hypothesis that Rs increases with increasing soil temperature in alpine grassland, but support the argument by Hibbard et al. [28] that within-site robust relationships with temperature and/or moisture are not adequate to characterize soil CO_2_ effluxes across space, because for regional variation BGB is the most important factor.

### Separating direct and indirect factors influencing soil respiration

In the present study, we used regression tree analysis [53] and SEM [56]–[58] as new approaches to conduct variable selection, to identify direct and indirect factors, and to determine the extent to which these factors may constrain Rs. To our knowledge, the efficiency of these approaches has not been evaluated empirically in soil respiration research.

Traditionally, stepwise selection and linear regression are used to identify and rank the limiting factors in Rs studies. However, when performing stepwise selection, closely covariated parameters cannot be selected simultaneously in the final model, because the explanatory power would not increase when a closely related variable is included. In our case, when BGB retain in the model, AGB will not be selected due to their close correlation. As a matter of fact, AGB has a strong indirect effect through BGB on Rs. This problem can be solved by a regression tree analysis which has the advantage to rank the limiting factors based on their importance [55].

Field studies examining ecosystem responses to climatic and other environmental changes typically use naturally occurring climatic gradients. However, some studies have realized the limitations of correlation method in analyzing factors influencing Rs [12], [76]. For example, Rs rates vary significantly among major plant biomes, suggesting that vegetation type influences the rate of soil respiration. Nevertheless, the correlations among climatic factors, vegetation distributions, and Rs make cause-effect arguments difficult [12]. Burke et al. [76] raised the issue that there are inherent problems with utilizing simple statistical relationships of spatial variability as a foundation for understanding ecosystem function, because complex covariance along the gradient occurs across large spatial scales, leading to the problem that actual and apparent controlling factors may be confounded. Without field experiments, which are difficult to conduct across numerous sites, and without simulation of ecological processes, which need to be based on mechanistic data, SEM is one option. The quantitative procedure in the current study showed that the direct factors influencing Rs at large-scale were BGB and SM, AGB, SOC and vegetation type only had indirect influences despite their significant correlations with Rs. This holistic approach is appropriate in across-site comparisons of ecosystem structure and function.

### Limitations of the current study

In the present study the soil PVC collars were installed only one hour before measurement due to the low accessibility of most sites, while the placement of collars are at least 24 hour prior to measurement in most Rs studies. Althouth the insertion of collars may cause unrealistic readings of soil CO_2_ efflux because of the high fluxes after colloar installation, fluxes stabilize after 10–30 min [77], [78]. In addition, our measurement of Rs followed the same procedure throughout our survey. Therefore the error introduced by soil disturbance could be treated as a systematic error which is weak.

Complete diurnal courses were obtained at nine sites, whereas for most of our sites soil respiration were measured 3–4 times during 4–5 hours when Rs peaked. We acknowledge that soil respiration is a dynamic process that may not be well represented by a few replicated measurements during several hours of a day. However, we found average midday Rs rates of the nine sites were well correlated with their daily mean Rs. Furthermore, we calculate daily mean Rs of each site by extrapolating the nine diurnal courses to all 42 sites according to community composition and closeness in distance. This extrapolation might add uncertainty to the estimates of daily mean Rs. Nevertheless, sites of similar vegetation composition and closest in distance generally share comparable features of geology, climate, soil and vegetation, which in combination are the major determinants of soil respiration.

The main objective of this study is to investigate the large-scale regional patterns of Rs in the Tibetan Plateau. Rs of 42 sites were measured during peak growing season of late July and early August. Measurements over a time span of one month may lead to problems as spatial variation of Rs could interfere with temporal changes. However, a four-year observation on soil CO_2_ efflux in Haibei Alpine Grassland Research Station of Northwest Institute of Plateau Biology, the Chinese Academy of Sciences (3200 m a.s.l.) revealed that Rs values peak and stabilize in late July and early August (unpublished data by YHW and JSH). This phenomenon was observed in north America as well [79]. Therefore, compared with the large variation of Rs across the plateau, the temporal interference should be minor.

### Conclusions and implications

Our understanding of the controls and magnitudes of regional Rs is limited by the uncertainties due to spatial heterogeneity of vegetation across regional environmental gradients. In the current study, we moved beyond within-site differences in soil temperature and moisture to incorporate differences among broad ecosystem types (e.g. biomes). We can conclude with certainty that BGB is the factor most closely associated with Rs rate at regional scale for the grassland ecosystems, suggesting that in future we could develop models for Rs from plant standing biomass, which has a much larger database with wider biogeographic coverage, particularly in remote areas, such as the Tibetan Plateau. We acknowledge that only Rs rates during peak growing season were measured in the current study. Therefore, intensive measurements should be taken on a few sites across environmental gradients to develop more precise prediction models for annual Rs. Our results also have the implication that if we take Rs rates at peak growing season as a parameter of ecosystem metabolic activity, then compared with the plant physiology at individual level, ecosystem metabolism is not so much influenced by temperature itself. Furthermore, our results imply that a shift from alpine meadow to steppe due to changes of soil hydrological properties as a consequence of permafrost degradation will significantly alter Rs.
